# 

**Published:** 1914-02-28

**Authors:** 


### Appointments.

Dr. F. Langmead has been appointed physician in charge of out-patients at St. Mary's Hospital, Paddington.

Mr. Joseph Harvey, M.B., B.Ch., has been appointed senior resident medical officer at Northampton General Hospital.

For the time being Dr. Tottenham has been appointed assistant house surgeon at the West Bromwich District Hospital.

Mr. Arthur H. Norris, M.R.C.S., L.R.C.P., has been appointed Medical Inspector of Reformatory and Industrial Schools by the Home Secretary from April 1.

Mr. Ernest Allan has been appointed fifth assistant medical officer at Hanwell Mental Hospital, in succession to Mr. Patrick Murphy, who has resigned.

Manchester Children's Hospital.—Dr. Arnold Jones, F.R.C.S.Edin., has been elected to the vacancy caused by the resignation of Mr. F. H. Westmacott, F.R.C.S., surgeon to the throat, nose and ear department for many years. Mr. Westmacott has been elected hon. consulting aural surgeon.

### Gifts and Bequests.

Mr. W. J. Tatem, a Cardiff shipowner, has given 1,000 guineas to endow a cot as a memorial of his mother at the Hamadryad Seamen's Hospital, Cardiff.

King Edward's Hospital Fund for London has received the sum of £1,000, the annual subscription of the King, who is patron and late president of the fund, and £105, the annual subscription of the Queen.

The Rev. W. F. Green, on behalf of the Budleigh Salterton Cottage Hospital, has received a promise of £500 for the building and equipment of an operation room from Mr. F. Williams, a local resident, in memory of his late wife.

The late Mr. William Raven, who in his lifetime was a generous contributor to the funds of Leicester Royal Infirmary, has bequeathed £1,000 to the infirmary, which the testator hopes will be invested. The late Miss Annie Harding, also of Leicester, has bequeathed £50 to the same institution.

Mrs. Marianne Costar, of Redhill, has left £5,000 each to St. Thomas's Hospital and Guy's; £2,000 each to the London, Charing Cross, Great Northern Central, Middlesex, Metropolitan, Kingsland Road, King's College, St. Mary's, University College, Westminster, Royal Free, and West London Hospitals, and the Redhill and Reigate Cottage Hospital; and £500 to the Royal Asylum of St. Anne's Society.

King Edward's Hospital Fund has received the following annual subscriptions: Mr. Walter Morrison, £5,000; Mr. William Waldorf Astor, £1,000; Messrs. C. J. Hambro and Son, £250; Prudential Assurance Co., Ltd., £250; Mrs. Walter Burns, £200; the Worshipful Company of Merchant Taylors, £1,000; Mr. Robert Fleming, £500; Messrs. Baring Brothers and Co., Ltd., £250; Lord Revelstoke, £100; Mr. George Lawson Johnston, £52 10s.; "I. J. H.," £50.

### LONDON SCHOOL OF TROPICAL MEDICINE.

Dr. R. T. Leiper, Helminthologist of the London School of Tropical Medicine and Wandsworth Scholar, has left London with Surgeon E. L. Atkinson, R.N. (who has been seconded by the Admiralty), and Mr. Cherry-Garrard, both of whom accompanied the Antarctic Expedition. The object of this expedition to the East is to ascertain the mode of spread of trematode diseases of man, especially bilharziasis; investigations will also be made into ankylostomiasis, which has wrought such havoc among the coolies on the tea and rubber estates. The investigators were seen off by Lady Scott, Mrs. Wilson, and others connected with the Antarctic Expedition.

Rates of Subscription: Twelve months, 6/6; Six months, 4/-; Three months, 2/-, post free to any address in the United Kingdom. Abroad, Twelve months, 8/8; Six months, 5/-; Three months, 2/6, post free.—Address The Subscription Dept., "The Hospital," The Hospital Building, 28/29 Southampton Street, Strand, London, W.C.

**28-29 Southampton Street, Strand, London, W.C.**

